# Establishment of a Veterinary School in Upper Austria

**Published:** 1896-09

**Authors:** 


Establishment of a Veterinary School in Upper Austria. At the session of the Upper Austria Landtag of January

29th, the following bill was passed: The House enacts and commissions the committee, which represents the Province, to enter into negotiations with the Imperial Government and work for the establishment of an institution for the training of farriers or horse-doctors, corresponding to the agricultural needs and circumstances, this school to be either in the Agricultural College at Ritzlhof or in any other place, provided that the institution be made accessible to people of only moderate means, and farriers be trained for the country who shall have the necessary practice, and who shall not be compelled to demand compensation which is too high and incompatible with the present agricultural conditions.

This bill was drafted by Representative Tannegger, and in speaking in behalf of the bill he said: "I picture the arrangement of the Farrier School at Ritzlhof, planned by me, about as follows: First, two or three veterinarians by profession as teachers, and twenty to forty blacksmiths who have finished their trade as pupils, the course to cover two years. Secondly, a hospital where sick animals from the neighborhood may be either cared for free or with only a small charge; and, thirdly, suitable buildings are to be erected for the smithy, school, and dwellings of the pupils."

The costs, he asserts, although pretty high, are nothing in comparison to the advantages which would accrue to the agricultural classes, and that the losses caused by empirics in one year would cover the cost of the institution three or four times.

The reasons urged for this school are: First, the training of a higher class of veterinarians in the school at Vienna, and their appointment to the larger provinces would be in no wise lessened; secondly, practical farriers whose livelihood is assured as blacksmiths would take the place of empirics; and, thirdly, quick and cheap assistance would be provided for the farmers, which would be a blessing to the cattle-breeding interests of the community.—*Thierärzt. Centralblatt*, May 1, 1896.

It is hardly necessary to remark that such a measure as this has aroused the opposition of qualified veterinarians and of all who have the real interests of the profession at heart. The *Centralblatt*, in an editorial of three pages and a half, shows the unreasonableness of the proposition, and urges the Imperial Government to set its veto upon it. In conclusion it says that the bill to which Representative Tannegger, an apothecary by profession, has the very doubtful honor of being the father, is plainly unreasonable. Instead of aiming at a reorganization of the veterinary service of the country in such a way that, by means of the provincial funds, veterinarians with fixed alaries may be appointed in all the principal places, as has been done already in the Crown Lands of Mähren, Lower Austria, Silesia, Bohemia, and elsewhere, these veterinarians doing the sanitary police work assigned to them by the Government, instead of establishing a fixed rate of compensation for veterinary services which all applicants for a position would be obliged to accept, instead of establishing adequate compensation, by means of which the youth of his province might obtain competent veterinary training; in short, instead of proposing anything that would accrue to the real interest of cattle-breeders and to veterinary science in general, Representative Tannegger thinks he has raised up a monument to himself in his bastard proposition.

